# Direct Isolation and Characterization of Human Nephron Progenitors

**DOI:** 10.5966/sctm.2015-0429

**Published:** 2016-09-09

**Authors:** Stefano Da Sacco, Matthew E. Thornton, Astgik Petrosyan, Maria Lavarreda‐Pearce, Sargis Sedrakyan, Brendan H. Grubbs, Roger E. De Filippo, Laura Perin

**Affiliations:** ^1^ GOFARR Laboratory for Organ Regenerative Research and Cell Therapeutics, Saban Research Institute, Division of Urology, Children's Hospital Los Angeles, Los Angeles, California, USA; ^2^ Maternal‐Fetal Medicine Division, Department of Obstetrics and Gynecology, Keck School of Medicine, University of Southern California, Los Angeles, California, USA; ^3^ Department of Urology, Keck School of Medicine, University of Southern California, Los Angeles, California, USA

**Keywords:** Human nephron progenitors, Smartflares, Direct isolation of live cells

## Abstract

Mature nephrons originate from a small population of uninduced nephrogenic progenitor cells (NPs) within the cap mesenchyme. These cells are characterized by the coexpression of SIX2 and CITED1. Many studies on mouse models as well as on human pluripotent stem cells have advanced our knowledge of NPs, but very little is known about this population in humans, since it is exhausted before birth and strategies for its direct isolation are still limited. Here we report an efficient protocol for direct isolation of human NPs without genetic manipulation or stepwise induction procedures. With the use of RNA‐labeling probes, we isolated SIX2^+^CITED1^+^ cells from human fetal kidney for the first time. We confirmed their nephrogenic state by gene profiling and evaluated their nephrogenic capabilities in giving rise to mature renal cells. We also evaluated the ability to culture these cells without complete loss of SIX2 and CITED1 expression over time. In addition to defining the gene profile of human NPs, this in vitro system facilitates studies of human renal development and provides a novel tool for renal regeneration and bioengineering purposes. Stem Cells Translational Medicine *2017;6:419–433*

**Significance Statement**

The use of RNA‐labeling probes allowed for the first time the definition of an efficient protocol for direct isolation of human nephron progenitors coexpressing SIX2 and CITED1, the master genes regulating renal development. These SIX2/CITED1‐positive cells were derived from fetal kidneys without the use of any reprogramming strategy or laborious stepwise induction protocols. Their nephrogenic state was confirmed by gene profiling, and their nephrogenic specification and culture conditions were evaluated. This first “snapshot” of the transcriptional network of human nephron progenitors opens new avenues in understanding human kidney development and nephron specification and supports the study's ultimate goal of understanding possible mechanisms for kidney regeneration.

## Introduction

In humans, approximately 500,000–1,000,000 nephrons are generated before the 34th to 36th week of gestational age (GA), a point at which nephrogenic progenitor cells (NPs) are fully exhausted and nephrogenesis ceases 1, 2. The loss of a sufficient number of nephrons at any time after this period leads to irreversible kidney failure, as no further nephrogenesis can occur.

The mammalian kidney, or metanephros, originates from the reciprocal interaction between two derivatives of the intermediate mesoderm: the metanephric mesenchyme (MM) and the ureteric bud (UB) 1. This complex process starts when the UB invades the MM, inducing the condensation of MM cells around the tips of the branching UB and giving rise to developing structures including pretubular aggregates, the renal vesicle, and comma‐ and s‐shaped bodies, with eventual formation of the mature nephron, consisting of the tubule and the glomerulus.

Within the cap mesenchyme (CM), a subdomain of the MM consisting of the cells closest to the UB tips and arranged in a series of compartments 3, resides a small pool of cells characterized by the expression of CITED1 (Cbp/p300‐interacting transactivator 1) and/or SIX2 (Sine oculis homeobox 2). Generation of promoter‐specific Cre mice and experiments of cell lineage tracing have shown that SIX2 defines the induced MM and is a critical regulator of the CM progenitor state. Notably, these studies have also confirmed that cells expressing both SIX2 and CITED1 are a true uninduced, nephron‐committed, multipotent, self‐renewing progenitor population that is capable of generating all the segments of the nephron 4, 5.

Importantly, most studies on NPs have been performed in murine models, specifically focusing on SIX2^+^ or CITED1^+^ cells; no study has yet described the direct isolation of mouse (or human) NPs based on the coexpression of both SIX2 and CITED1 1. Significant studies using human embryonic stem cells (ESCs) 6, induced pluripotent stem cells (iPSs) 6, 7, or direct reprogramming to NPs 8 have proved that it is possible to obtain cells expressing renal developmental genes.

More recently, cutting‐edge publications have demonstrated that iPSs can be guided through stepwise protocols to form renal organoids with high efficiency 9, 10, 11. Nevertheless, these studies were focused on the derivation of NPs from iPSs, mostly targeting the inducible SIX2‐positive cells, and no characterization was reported about the uninduced NPs (SIX2^+^CITED1^+^ NPs).

In the current work, we report for the first time the direct isolation of a population of cells expressing both *SIX2* and *CITED1* from human fetal kidney (hFK), combining the use of a fluorescent RNA probe technology with fluorescence‐activated cell sorting (FACS). After validation of this technique, we characterized this population in terms of gene profiling by RNA sequencing (RNA‐seq), evaluated their expansion in vitro, and tested their in vitro nephrogenic capability. We also compared this population with mouse nephron progenitors in terms of gene expression.

The protocols established in this study allowed the first characterization of human NPs coexpressing SIX2 and CITED1 obtained from an endogenous source, specifically without the use of any reprogramming or induction procedures. This opens new avenues in understanding human kidney development and nephron specification and formation and supports our ultimate goal of understanding possible mechanisms for kidney regeneration.

## Materials and Methods

### Acquisition of hFK Samples

hFK tissue collection was approved by the institutional review boards of both Children's Hospital Los Angeles and the University of Southern California, and samples were obtained from the Children's Hospital Los Angeles Tissue Bank. Twenty‐six samples of hFK (approximately 17 weeks GA) were used to perform all the experiments; specifically, 10 samples were used for cell isolation, 3 samples for RNA‐seq, 3 samples for staining of live renal slices, 3 for immunohistochemistry and immunofluorescence analysis, 5 for dissociation/reaggregation experiments, and 2 for RNA and protein extraction. After digestion with 0.05% collagenase I (BD Biosciences, San Jose, CA, http://www.bdbiosciences.com) at 37°C for 90 minutes and elimination of erythrocytes by Blood Lysis kit (Miltenyi Biotec, Cambridge, MA, http://www.miltenyibiotec.com), single‐cell suspensions from hFK were obtained.

### Smartflare RNA Probe Isolation and Culture of SIX2^+^CITED1^+^ Cells

hFK single‐cell suspension was incubated overnight with both SIX2‐cyanine 5 (Cy5) and CITED1‐Cy3 Smartflare RNA probes (SF‐1075 and SFC‐319, respectively; EMD Millipore, Billerica, MA, http://www.emdmillipore.com) following the manufacturer's instructions. Briefly, RNA probes were diluted 1:20 in phosphate‐buffered saline and 25 µl/ml was added to the culture medium. Scrambled probes (negative control) and uptake probes (positive control) were used across all the experiments. After FACS, cells were in Chang medium 12 or RMPI 1640, 10% fetal bovine serum (FBS), and 1% antibiotic (Thermo Fisher Scientific Life Sciences, Waltham, MA, http://www.thermofisher.com); cells were passaged using 0.05% trypsin‐0.01% EDTA (Thermo Fisher). hAKPC‐P cells at passage 15–20 were isolated and cultured as described 12.

### RNA‐Seq Experiments

RNA extraction was performed immediately after FACS (passage 0) using the RNeasy Micro Kit (Qiagen, Valencia, CA, http://www.qiagen.com) following the manufacturer's recommendations. After cDNA production (manufacturer's protocol; Clontech, Mountain View, CA, http://www.clontech.com) and construction of DNA libraries, the samples were run on an Illumina NextSep500 (Illumina, San Diego, CA, http://www.illumina.com). Differential gene expression was analyzed using ERCC ExFold probes with the Remove Unwanted Variation R/Bioconductor software package 13 combined with edgeR 14. Gene ontology enrichment analysis was performed using GOstats R/Bioconductor software 15. A detailed description of the RNA‐seq method and data analysis is provided in the supplemental online data. Data have been deposited in Gene Expression Omnibus (GEO) under accession number GEO: GSE74450.

### Polymerase Chain Reaction Analysis, Histochemistry, Immunofluorescence, Western Blot, and FACS

RNA extraction and polymerase chain reaction analysis, immunostaining, hematoxylin and eosin staining, and FACS sorting were performed as previously described using standard protocols 12, 16, 17, 18, 19. Renal slices for staining of live tissue were obtained by hFK agarose embedding following a protocol adapted from standard procedures 20. After embedding, 300‐µm slices were cut with the use of a vibratome (Leica Microsystems, Buffalo Grove, IL, http://www.leica‐microsystems.com). Slices were transferred in 48‐well plates and stained with Smartflare RNA probes as described above. Apoptosis in 10% formalin‐fixed cells was evaluated by BAX staining in cells cultured with 5 ng/ml tumor necrosis factor‐α for 6 hours (positive control) and in negative controls (untreated). Antibody concentrations and primers are described in the supplemental online data. Western blot for α3 and α5 chains was performed following the protocols described in Sugimoto et al. 21 and previously adapted by our group 12, 19. Briefly, under reducing conditions, protein extracts were separated on 4%–20% Tris‐Glycine gel (Thermo Fisher) and transferred onto a polyvinylidene fluoride 0.45‐μm membrane. Blotted membranes were blocked with 3% bovine serum albumin containing 50 mM Tris‐HCl buffer (pH 7.5) and 150 mM NaCl for more than 8 hours, washed 3 times with 0.1% Tween‐Tris buffer, treated with primary type IV collagen antibodies for 2 hours, and diluted 1:100 in 1% bovine serum albumin containing 50 mM Tris‐HCl buffer (pH 7.5) and 150 mM NaCl (H31, H52; Shigei Medical Research Institute, Okayama, Japan). Horseradish peroxidase‐conjugated secondary antibody was applied thereafter. Detection of antigens was performed using ECL Western blotting detection reagents (GE Healthcare, Waukesha, WI, http://www.gehealthcare.com), impressed on Biomax Light Film (GE Healthcare).

### Clone Generation

Clones were obtained by limiting dilution immediately after sorting (passage 0). Briefly, 300–400 cells were singly plated in each well of 96‐multiwell plates. Four plates were prepared for each hFK‐derived sample. Cultures were examined daily for the appearance of colonies. Wells containing more than one colony were not considered. All the clones that reached confluence were detached with 0.05% trypsin‐0.01% EDTA (Sigma‐Aldrich, St Louis, MO, http://www.sigmaaldrich.com), and each of them was plated in 4 wells (replicas) of a 24‐multiwell plate.

### Dissociation/Reaggregation Assays

hFK cells were mixed in a 10:1 ratio with hFK‐derived SIX2^+^CITED1^+^ cells, at passage 5 after selection, previously labeled with CM‐DiI (Thermo Fisher) following standard protocols 17, 19. Cells were transferred onto polycarbonate membrane (3‐µm pore size) at the air‐liquid surface in Dulbecco's modified Eagle's growth medium in a 24‐well plate for 7 days. After 7 days of culture, the kidney explants were fixed with 4% PFA. CM‐Dil‐labeled cells were visualized by immunofluorescence microscopy after immunostaining.

### Induction, 3D Collagen Experiments, and Podocyte Induction

Induction of hFK cells toward differentiation was performed by adding Wnt9b (0.4 μg/ml) and bone morphogenetic protein (BMP)‐7 (0.05 μg/ml) to the culture medium for 7 days. Cells were then harvested and fixed, and flow cytometry analysis was performed to evaluate expression of SIX2 and CITED1 as previously described 12.

Induction into tubular‐like cells was performed by seeding the cells at passage 5 after selection into a 3‐dimensional (3D) collagen layer using the 3D collagen assay kit (EMD Millipore), following the manufacturer's instructions. Cells were placed into 24‐well plates and cultured for up to 21 days with RPMI 1640, 10% embryonic stem FBS, and 1% penicillin‐streptomycin. Additionally, in a parallel experiment, SIX2^+^CITED1^+^ cells were induced toward a renal tubular fate by stimulation with BMP‐2 and BMP‐7 as described by Narayanan et al. 22 for 14 days. Podocyte induction was performed as previously published 12. Briefly, differentiation was performed by culturing the cells on collagen I‐coated plates in VRADD media (RPMI‐1640 supplemented with 10% FBS, 1% antibiotic, 100 nM 1,25(OH)_2_D_3_, 1 µM all‐trans retinoic acid, 100 nM dexamethasone, and 1× insulin‐transferrin‐selenite) for up to 30 days.

### Maintenance of Nephrogenic Markers

Maintenance of expression of nephrogenic markers was tested (at different passages in culture or immediately after isolation) in SIX2^+^CITED1^+^ cells cultured in Chang medium (control group) or nephrogenic progenitor expansion medium (NPEM) 23. Immunofluorescence and flow cytometry analysis were performed to evaluate SIX2 and CITED1 messenger RNA (mRNA) and protein expression after 7, 15, and 21 days in culture (passages 3, 6, and 10, respectively). To reflect the differences in cell size and granularity between the experimental groups caused by different culture conditions and time points, gates were drawn for each assay independently to reflect those differences and avoid inconsistencies in the measurements of positive and negative fractions. The gating strategy is described in supplemental online Fig. 4.

## Results

### Isolation of SIX2^+^CITED1^+^ NPs From hFK and Smartflare Validation

The direct isolation of SIX2^+^ or CITED1^+^ NPs until now has relied on the use of transgenic mice with fluorescence‐tagged NPs, as these two proteins are localized within the intracellular compartment. Some reports have identified the presence (or absence) of surface markers that allow the isolation of live cells expressing SIX2 and/or CITED1 in mice 24, 25. One report by Harari‐Steinberg et al. 26, and more recently another publication from the same group 27, proposed neural cell adhesion molecule 1 as a reliable cell surface marker for the isolation of cells expressing SIX2 from hFK 26, therefore identifying human renal progenitors; however, the expression of CITED1 was not reported in the isolated cells.

**Figure 1:**
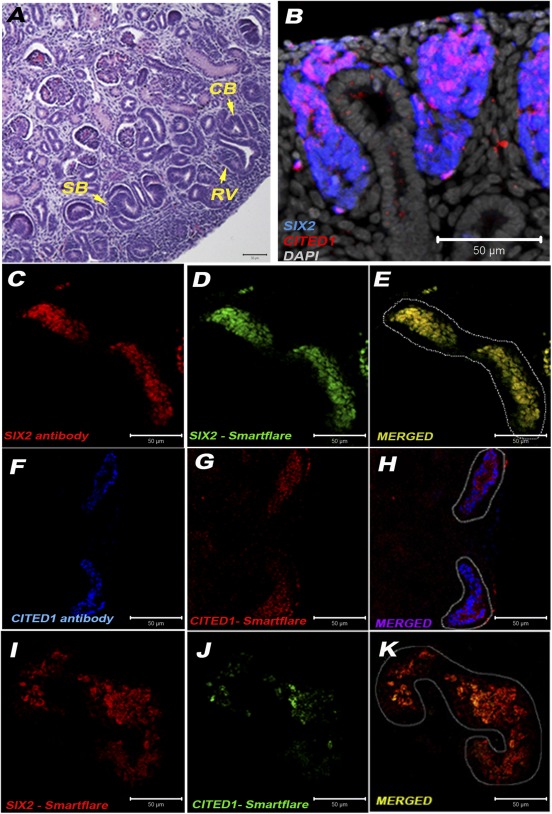
Colocalization of SIX2^+^CITED1^+^ cells within human fetal kidney (hFK). **(A):** Within hFK (17 weeks gestational age [GA]), nephrogenic structures are recognizable, including V, SB, and CB (arrow) (magnification, ×20; scale bar = 50 μm). **(B):** Immunostaining showing expression of SIX2 (blue) in cells within the MM and colocalization (magenta) of SIX2 and CITED1 (red) in cells within the CM in close proximity of ureteric bud (UB) branching as well as along the edges of the UB (magnification, ×20; nuclei stained gray, DAPI; scale bar 50 μm). **(C–K):** Cellular coexpression in agarose tissue sections (magnification, ×20; scale bar = 50 μm) of SIX2 antibody **(C)** (red) and SIX2‐Cy5 probe **(D)** (green) is shown in yellow **(E)**, coexpression of CITED1 antibody **(F)** (blue) and CITED1‐Cy3 probe **(G)** (red) is shown in purple **(H)**, and coexpression of SIX2‐Cy5 probe **(I)** (red) and CITED1‐Cy3 probe (green) **(J)** is shown in yellow **(K)**. Abbreviations: CB, comma‐shaped bodies; SB, s‐shaped bodies; RV, renal vesicles.

In an attempt to find alternative approaches for the isolation of pure NPs, we evaluated the efficiency of Smartflare fluorescent‐tagged RNA probes 28, 29, specifically designed to recognize SIX2 and CITED1 mRNA in live cells (SIX2‐Cy5 and CITED1‐Cy3). First, we confirmed the presence of SIX2^+^CITED1^+^ NPs within the CM by immunofluorescence in hFK at 17 weeks GA (Fig. 1A, 1B). Interestingly, as evident in Figure 1B (and all the following immunostaining), CITED1 expression in human CM extends further below the tip of the UB branching. To validate our technique, we showed that SIX2‐Cy5 and CITED1‐Cy3 probes identify the NPs directly within the CM of hFK agarose‐embedded live tissue (Fig. 1C–1K; supplemental online Video 1). Notably, with both Smartflare and antibody staining it is possible to identify the existence of both a SIX2^+^CITED1^+^ (uninduced) and a SIX2^+^CITED1^‐^ (induced) population as expected 1. SIX2 and CITED1 stained by either RNA probes or antibody colocalized, suggesting specificity of the Smartflare approach. In addition, the signal for SIX2‐Cy5 and CITED1‐Cy3 probes is present only in cells within the MM (in proximity of the UB branching, as expected), therefore clearly indicating the specificity of the probes for the uninduced NP population (supplemental online Fig. 1A–1C).

**Figure 2:**
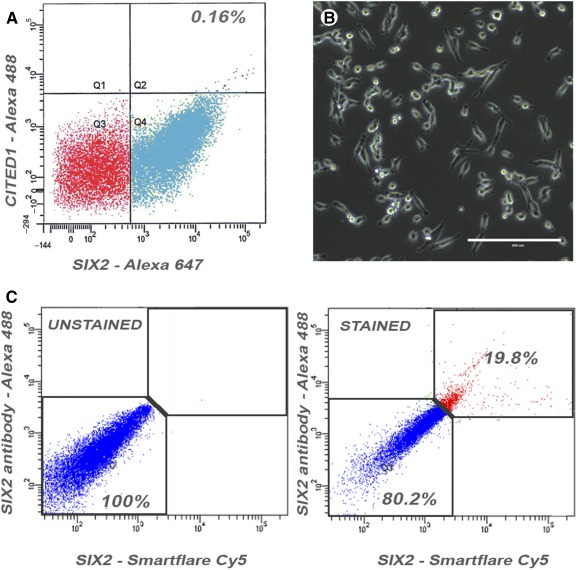
Isolation of SIX2^+^CITED1^+^ cells from human fetal kidney (hFK) and validation of SIX2‐Cy5 and CITED1‐Cy3 probe technique. **(A,B):** Smartflare‐isolated SIX2^+^CITED1^+^ cells represent approximately 0.16% of the total hFK cell population and display a fibroblastoid morphology (**B**, scale bar = 400 μm; magnification, ×20). **(C):** Flow cytometry analysis of SIX2^+^ cells selected using SIX2 antibody (Alexa‐Fluor 488) and SIX2‐Cy5 probe showing that 19.8% of the cells are double positive for both markers and 80.2% of the cells are negative for both markers, whereas extremely low number of cells are single positive for either SIX2‐Smartflare or the SIX2 antibody. Abbreviations: Cy, cyanine.

After dissociation of hFK into single‐cell suspension and incubation with SIX2‐Cy5 and CITED1‐Cy3 followed by FACS sorting, an average of 0.16% of the total cells were found to be SIX2^+^CITED1^+^ (Fig. 2A). These cells had a fibroblastoid morphology immediately after plating (Fig. 2B). Importantly, we validated the Smartflare technique by flow cytometry analysis using both SIX2 antibody and SIX2‐Cy5 probe; indeed, Figure 2C confirms the specificity of probe and antibody by costaining the same population. In addition, we confirmed the overlapping expression of SIX2‐Cy5 and CITED1‐Cy3 with SIX2 and CITED1 proteins by immunostaining after 24 hours in culture. This process established that, after isolation, the mRNA probes were present in cells that also expressed SIX2 and CITED1 proteins, thus further validating this technique in vitro (supplemental online Fig. 1D–1F). The Smartflare labeling did not affect cell viability, as indicated by evaluation of apoptosis (Bax staining) performed in Smartflare‐labeled cells and compared with cells treated with tumor necrosis factor‐α (a known inducer of apoptosis, positive control) and untreated, unlabeled cells (negative control) (supplemental online Fig. 1G, 1H).

To further validate the Smartflare technique and evaluate its versatility, we also investigated the possibility of isolating SIX2^+^CITED1^+^ cells from an exogenous source and comparing them with fibroblasts (negative control). We chose to focus our attention on the amniotic fluid (AF), as our group has extensive experience in the isolation of different cell types from AF and the evaluation of their potential for kidney regeneration 12, 17, 18, 19, 30.

We have previously identified a population of cells (hAKPC‐P; supplemental online Fig. 2A) selected for CD24, OB‐Cadherin, and podocalyxin within hAF that can be differentiated in vitro into podocyte‐like cells 12. Smartflare labeling was performed on hAKPC‐P cells, and we successfully reported the isolation of SIX2^+^CITED1^+^ cells (0.2%–0.3%) as shown in the hFK and confirmed the specificity of the probes for this source of cells (supplemental online Fig. 2B–2F). Fibroblasts did not stain for the probe (supplemental online Fig. 2G). These data validate the use of Smartflare as an efficient method to isolate NPs from different sources without transfection and with no cellular damage.

### Gene Profiling in SIX2^+^CITED1^+^ Cells From hFK and Comparison With Mouse NPs

**Figure 3:**
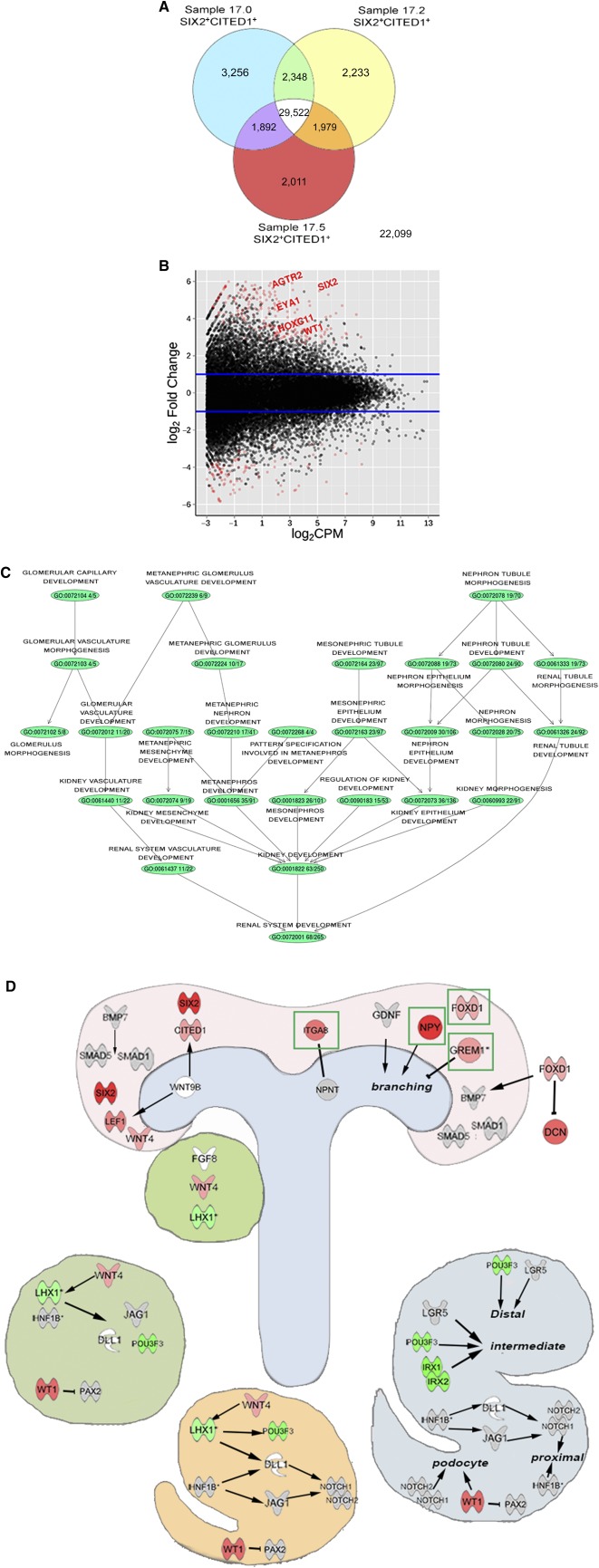
Gene profiling of SIX2^+^CITED1^+^ cells from human fetal kidney (hFK). **(A):** Venn diagram showing coexpression of genes in SIX2^+^CITED1^+^ cells isolated from the 3 samples of hFK (17.0, 17.2, and 17.5 weeks gestational age). **(B):** Smear plot representation of differentially expressed genes in SIX2^+^CITED1^+^ cells compared with hFK negative fraction. Blue lines indicate 1.5‐fold increase or decrease. **(C):** Gene set enrichment analysis for renal system development compartments including metanephros development (35/91), metanephric mesenchyme development (7/15), metanephric nephron development (17/41), and metanephric glomerulus development (10/17). Colored circles (nodes) represent gene ontology‐associated genetic pathways that were significantly enriched in the SIX2^+^CITED1^+^ cell fraction. **(D):** Ingenuity pathway analysis for key genes involved in different phases of nephrogenesis, including cap mesenchyme, ureteric bud, pretubular aggregates, renal vesicles, and comma‐ and s‐shaped bodies. Red genes were significantly overexpressed in SIX2^+^CITED1^+^ cells compared with the negative fraction, green genes were significantly underexpressed in SIX2^+^CITED1^+^ cells compared with the negative fraction, gray genes were not significantly differently expressed, and white genes were not expressed.

We performed RNA‐seq on 3 samples of hFK kidneys at 17 weeks GA (supplemental online Fig. 3A) and compared the SIX2^+^CITED1^+^ cells with their negative fraction (a heterogeneous population including SIX2^+^CITED1^‐^ cells and SIX2^‐^/CITED1^‐^ cells). As shown in Figure 3A, of 65,340 genes, 29,522 were expressed by all 3 samples, whereas 22,099 genes were not expressed at all in the positive populations. On the other hand, each cell line uniquely expressed 2,000–3,200 genes while sharing approximately 2,000 genes with one but not the other population.

RNA‐seq analysis upon remove‐unwanted‐variation normalization (supplemental online Fig. 3B) comparing gene expression between the SIX2^+^CITED1^+^ cells with the negative fraction revealed 4,224 differentially expressed genes, including genes involved in nephrogenesis such as *SIX2*, *WT1*, *EYA1*, and *HOX* paralogs (logFC > 1.5 ≤ 1.5; *p* < .05), of which 1,640 were overexpressed and 2,584 were underexpressed (Fig. 3B; supplemental online Table 1).

**Figure 4:**
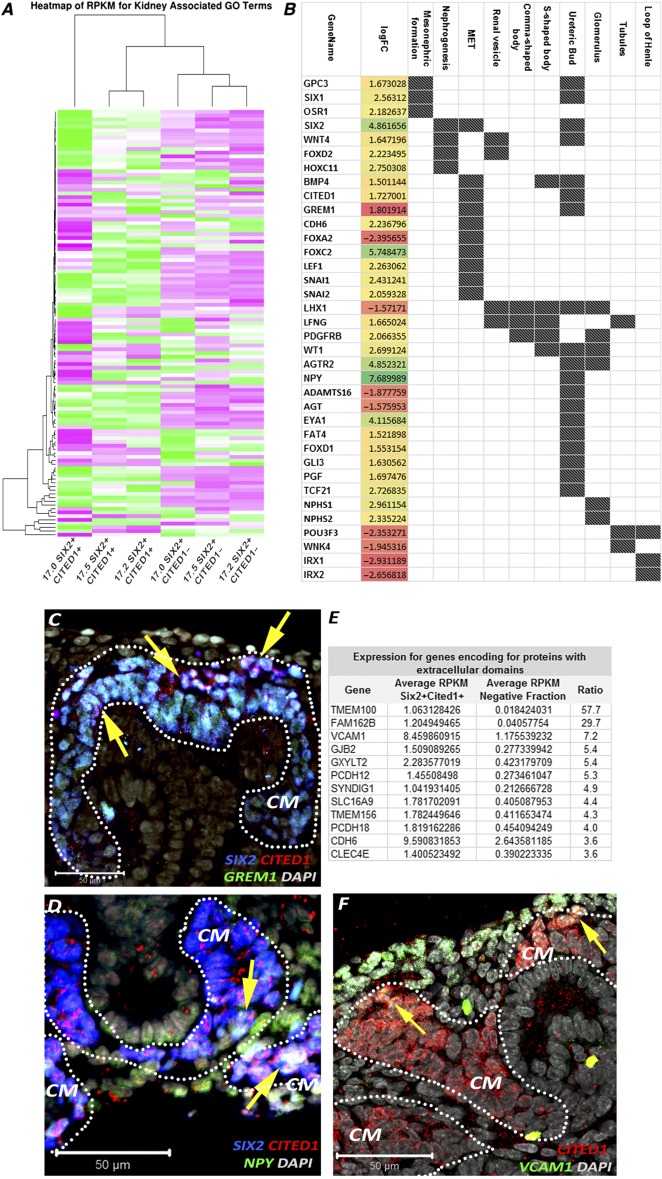
Differential gene expression in SIX2^+^CITED1^+^ cells from human fetal kidney (hFK). **(A):** Heat map showing relative expression (measured in RPKM) in SIX2^+^CITED1^+^ cells from hFK and negative fraction for genes involved in nephrogenesis, induction, specification, and differentiation. **(B):** Differentially expressed genes (logFC, *p* < .05) involved in nephron development in SIX2^+^CITED1^+^ cells (specific renal developmental processes represented by dark spot) compared with hFK negative fraction. **(C–D):** Confocal images showing colocalization (arrows) of staining for SIX2 (blue), CITED1 (red), GREM1 **(C)** (green), and NPY **(D)** (green) in cells in the metanephric mesenchyme in hFK (17 weeks gestational age [GA]; magnification, ×40; nuclei stained gray, DAPI; scale bar = 50 μm). **(E):** List of genes highly expressed in the SIX2^+^CITED1^+^ cell fraction and encoding for proteins with extracellular domains. **(F):** Confocal image showing colocalization (arrows) of staining for CITED1 (red) and VCAM1 (green) in hFK (17 weeks GA; nuclei stained gray, DAPI; magnification, ×40; scale bar = 50 μm). Abbreviations: CM, cap mesenchyme; DAPI, 4′,6‐diamidino‐2‐phenylindole; GO, gene ontology; logFC, log(fold‐change); MET, mesenchymal‐to‐epithelial transition; RPKM, reads per kilobase of transcript per million mapped reads.

After gene ontology analysis, we confirmed a statistically significant enrichment for genes playing a role in renal system development (66/265, such as *HOX* genes, *LHX1*, and *LEF1*) and in particular, a significant presence of genes involved in metanephros development (35/91, such as *EYA1*, *FOXD1*, and *FBN1*), mesenchyme development (9/19, such as *FOXC2*, *SIX2*, and *MEOX1*), and MM development (7/15, such as *OSR1*, *SIX1*, and *WT1*), confirming the MM origin of the isolated NPs (Fig. 3C, 3D). Along with genes usually recognized as key players in mouse kidney development, including *EYA1*, *HOX* genes, and *SALL1* (Fig. 4A, 4B), our analysis revealed that genes such as *SNAI2*, *FOXC2*, *SIX1*, *NPY*, and *GREM1* were highly expressed in our isolated populations. We also established the specific localization of NPY and GREM1 within the CM and their coexpression with the SIX2^+^CITED1^+^ cells (Fig. 4C, 4D), thus confirming their role during human kidney development.

When comparing the SIX2^+^CITED1^+^ cells to the remainder fraction of cells, we identified more than 300 (ratio positive/negative >1.5) genes encoding for proteins with extracellular domains that are highly expressed by the SIX2^+^CITED1^+^ cells, such as *TMEM100* (57×), *VCAM1* (7×), *GJB2* (5.4×), *GXYLT2* (5.3×), and *PCDH12* (5.4×) (Fig. 4E; supplemental online Table 2), and we confirmed the presence of VCAM1 in the SIX2^+^CITED1^+^ cells (Fig. 4F).

**Figure 5:**
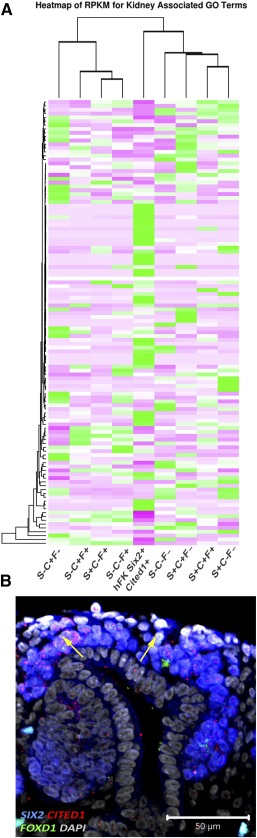
Comparison of gene expression between SIX2^+^CITED1^+^ cells from hFK and mouse nephrogenic progenitor cells (NPs). **(A):** Heat map showing relative expression (RPKM) of renal development‐related genes in SIX2^+^CITED1^+^ cells from hFK compared with S, C, and F, because NPs from hFK express FODX1 by RNA‐sequencing (RNA‐seq). GEO Dataset: GSE59127. For the purpose of comparing RNA‐seq data between humans and mice, single‐cell analyses were stratified by expression for *SIX2*, *CITED1*, and *FOXD1.* Specifically, mouse cells were grouped as follows: (1) SIX2^+^CITED1^+^FOXD1^+^, (2) SIX2^+^CITED1^+^FOXD1^–^, (3) SIX2^+^CITED1^–^FOXD1^–^, (4) SIX2^–^CITED1^–^FOXD1^–^, (5) SIX2^+^CITED1^+^FOXD1^–^, (6) SIX2^–^CITED1^+^FOXD1^–^, (7) SIX2^–^CITED1^+^FOXD1^+^, and (8) SIX2^–^CITED1^–^FOXD1^+^. **(B):** Immunostaining of hFK (17 weeks GA) confirming coexpression of SIX2 (blue), CITED1 (red), and FOXD1 (green) in cells in the metanephric mesenchyme (nuclei stained gray, DAPI; magnification, ×40; scale bar = 50 μm). Abbreviations: C, murine metanephric mesenchyme single cells stratified by expression of CITED1; DAPI, 4′,6‐diamidino‐2‐phenylindole; F, murine metanephric mesenchyme single cells stratified by expression of FOXD1; GA, gestational age; GEO, Gene Expression Omnibus; GO, gene ontology; hFK, human fetal kidney; RPKM, reads per kilobase of transcript per million mapped reads; S, murine metanephric mesenchyme single cells stratified by expression of SIX2.

In an attempt to understand the degree of similarity between human SIX2^+^CITED1^+^ cells and murine cells from the CM, we compared hFK‐derived NPs with genome‐wide analysis previously performed on single‐cell analysis by Brunskill et al. 31. After stratification of the samples based on gene expression, we compared hFK NPs for renal progenitor markers as shown in Figure 5A. hFK expressed higher relative levels of genes such as *WWTR1*, *CD44*, *MYC*, *FOXC2*, *PLCE1*, and *BASP1* (known for their important role during nephron development 32) and, interestingly, high levels of *SIX1* and *FOXD1*, possibly confirming an important role for these genes in human nephrogenesis 33. Remarkably, we confirmed the presence of FODX1^+^ cells in the CM SIX2^+^CITED1^+^ cells (Fig. 5B) 34. It is important to note that the isolation method for these cells differs from the Smartflare technique used in the current work. Nevertheless, although a more comprehensive analysis is needed, we think that this preliminary comparison highlights that human NPs might present a different gene expression than that of mice, possibly opening new insights into human renal development and repair. Importantly, these observations are supported by other publications in which differences in gene expression between human and mouse NPs was reported 2, 34, 35.

### Expansion, Clone Derivation, Maintenance of SIX2 and CITED1 Expression in Culture, and Renal Induction in SIX2^+^CITED1^+^ Cells From hFK

As previously described, although the isolation of NPs from embryonic mouse kidney by mechanical means 24, 25 or the derivation of NPs by differentiation of iPSs, ESCs, or reprogramming 7, 9 is feasible, the expansion of these cells and the maintenance of both SIX2 and CITED1 expression in selected populations is still hard to achieve with high reproducibility, particularly for human NPs. In fact, only one report, by Oxburgh's group 23, has established a protocol in which mouse CITED1^+^ cells can be propagated in vitro maintaining both SIX2 and CITED1 expression for at least 10 passages as confirmed by immunostaining.

When analyzing hFK NPs for the expression of a panel of stem cell markers, including self‐renewal genes such as *OCT4* and *NANOG*, we found that this population indeed expressed pluripotent genes (supplemental online Fig. 4A). Therefore, to test pluripotency and self‐renewal, we evaluated the clonal efficiency of NPs immediately after RNA probe selection (passage 0; supplemental online Fig. 4B). Specifically, the clonal efficiency for hFK cells was 7%–10%. Indeed, we confirmed that the clonal populations were still expressing EYA1, SALL1, and HOXA11, in addition to SIX2 and CITED1, at passage 3 (supplemental online Fig. 4C).

**Figure 6:**
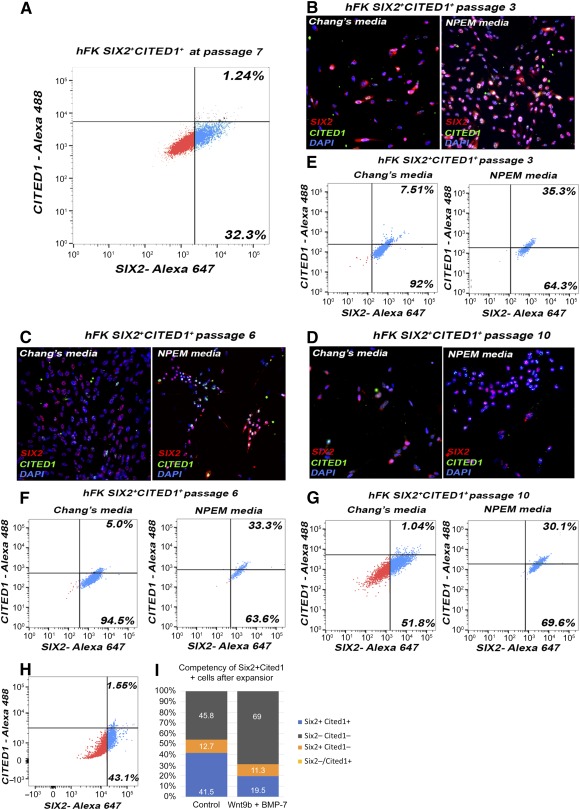
Expansion and maintenance of SIX2 and CITED1 expression in culture in SIX2^+^CITED1^+^ cells from hFK. **(A):** Fluorescence‐activated cell sorting (FACS) analysis of SIX2^+^CITED1^+^ cells from hFK showing loss of expression (hFK: 1.24%) after 7 passages in Chang medium. **(B–D):** Immunostaining for SIX2 (red) and CITED1 (green) in cells from hFK showing that coexpression is sparse after culture in Chang medium but is maintained in culture with NPEM at 3 **(B)**, 6 **(C)**, or 10 **(D)** passages (nuclei stained blue, DAPI; magnification, ×20). **(E–G):** FACS analysis of SIX2^+^CITED1^+^ cells from hFK showing maintenance at passage 3 **(E)**, 6 **(F)**, and 10 **(G)** of SIX2 and CITED1 in NPEM compared with the culture in Chang medium. **(H):** FACS analysis of SIX2^+^CITED1^+^ cells from hFK showing that NPEM medium does not reestablish expression of SIX2 and CITED1 in cultured cells previously expanded in Chang medium for 7 passages. **(I):** FACS analysis showing that administration of WNT9b and BMP7 for 5 days leads to a marked loss of SIX2 and CITED1 expression, suggesting that cells upon culture can be induced toward renal specification while losing self‐renewing nephrogenic capability. Abbreviations: DAPI, 4′,6‐diamidino‐2‐phenylindole; hFK, human fetal kidney; NPEM, nephrogenic progenitor expansion medium.

Even when we were able to culture NPs from hFK and derive clones, the expansion of these cells resulted in the loss of expression of SIX2 and CITED1 protein over time, as confirmed by previous studies 25. After 7 passages, flow cytometry analysis showed that the SIX2^+^CITED1^+^ population decreased to 1% and the SIX2^+^CITED1^‐^ cells were approximately 30%–40%, with the remaining fraction being negative for both markers (supplemental online Fig. 4D; Fig. 6A), suggesting differentiation in vitro.

The NPEM medium developed by Oxburgh's group 23 was formulated to promote the expansion and maintenance of SIX2 and CITED1 expression in both mouse NPs and human iPS‐derived NPs. NPs cultured in NPEM showed higher SIX2 and CITED1 expression compared with those cultured in Chang medium, as confirmed after 7 days (passage 3; Fig. 6B, 6E), 15 days (passage 6; Fig. 6C, 6F), and 21 days (passage 10; Fig. 6D, 6G) in culture. Importantly, we confirmed by flow cytometry analysis the maintenance of SIX2 and CITED1 expression after 21 days. This suggests that NPEM is indeed a suitable medium for NP expansion, but only when cells are cultured in NPEM immediately after RNA probe selection; in fact, NPEM was unable to rescue expression of SIX2 and CITED1 in cells previously expanded in Chang medium, suggesting that the in vitro culture conditions after isolation lead to changes in gene expression (Fig. 6H).

**Figure 7:**
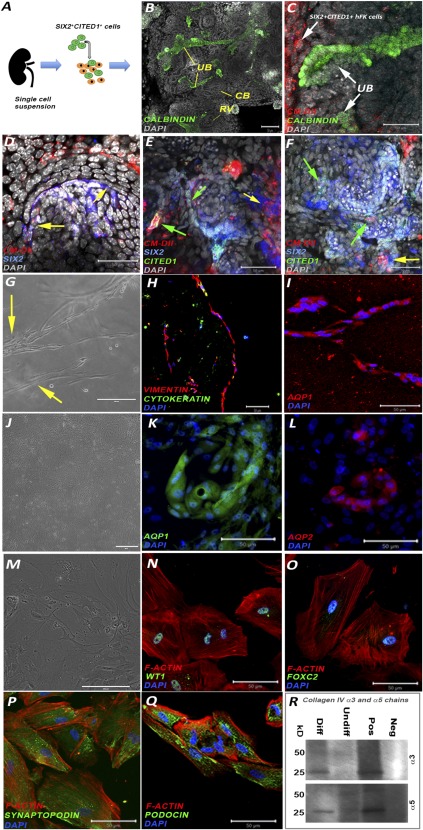
SIX2^+^CITED1^+^ cells from hFK: ex vivo organoids and in vitro induction. **(A):** Scheme representing the ex vivo organoid experiment. **(B):** Immunostaining of calbindin (green) identifying UB structures (arrow) of a reaggregated hFK organoid after 7 days in culture. The organoids reorganized forming all the typical structures including RV and CB. bodies. (Nuclei stained gray, DAPI; magnification, ×20; scale bar = 50 μm.) **(C,D):** Confocal images of CM‐Dil‐labeled SIX2^+^CITED1^+^ cells (red) from hFK showing localization of labeled cells in proximity to the UB, identified by calbindin staining (green, arrow) **(C)** and colocalization (purple, arrows) **(D)** staining with SIX2 (blue) within the cap mesenchyme. (Nuclei stained gray, DAPI; magnification, ×20; scale bar = 50 μm.) **(E,F):** Confocal images of CM‐Dil‐labeled SIX2^+^CITED1^+^ cells (red) from hFK showing localization of labeled cells with SIX2 and CITED1 (green arrows) or SIX2 (yellow arrows). (Nuclei stained gray, DAPI; magnification, ×20; scale bar = 50 μm.) **(G–I):** Immunostaining of SIX2^+^CITED1^+^ cells from hFK showing the formation of tubule‐like structures (arrow) (magnification, ×10; scale bar = 200 μm) **(G)** when cultured in a three‐dimensional collagen layer for up to 7 days with coexpression of vimentin (red, mesenchymal marker) and cytokeratin (green, epithelial marker) (magnification, ×10; scale bar = 50 μm) **(H)** and expression of AQP1 (red) (magnification, ×20; scale bar = 50 μm) **(I)**. (Nuclei stained blue, DAPI; magnification, ×20; scale bar = 50 μm.) **(J–L):** Immunostaining of SIX2^+^CITED1^+^ cells from hFK upon induction toward tubular differentiation by addition of bone morphogenetic protein‐2 and ‐7 to the culture media. Although a variety of different structures are present **(J)** (magnification, ×4; scale bar = 100 μm), we confirmed the existence of partially organized tubule‐like structures expressing AQP‐1 (green) **(K)** and AQP‐2 (red) **(L)**. (Nuclei stained blue, DAPI; magnification, ×20; scale bar = 50 μm.) **(M–Q):** Immunostaining of SIX2^+^CITED1^+^ cells from hFK after podocyte differentiation showing arborized morphology and numerous primary processes **(M)** and expression of WT1 (green) **(N)**, FOXC2 (green) **(O)**, synaptopodin (green) **(P)** and podocin (green) **(Q)**. Phalloidin staining (red) identifies the actin cytoskeleton. (Nuclei stained blue, DAPI; magnification, ×20; scale bar = 50 μm. **(R):** Western blot analysis confirmed protein expression of collagen IV α3 and α5 chains in differentiated cells compared with undifferentiated cells and fibroblasts (negative control). Positive control: human kidney (25 kD, monomeric form; 50 kD, dimeric form). Abbreviations: CB, comma‐shaped bodies; CM, cap mesenchyme; DAPI, 4′,6‐diamidino‐2‐phenylindole; Diff, differentiated; hFK, human fetal kidney; Neg, negative control; RV, renal vesicles; Pos, positive control; UB, ureteric bud; Undiff, undifferentiated.

Because NPs lose the expression of SIX2 and CITED1, we performed initial experiments to evaluate whether NPs isolated from hFK present nephrogenic induction and specification. The ability to be induced in vitro was confirmed by loss of CITED1 in SIX2^+^CITED1^+^ cells after exposure to BMP‐7 and Wnt9b, known to be the dominant signal‐initiating exit of NPs from their state of self‐renewal and inducing differentiation into epithelial renal cells 1 (Fig. 6I). Furthermore, CM‐DiI‐labeled SIX2^+^CITED1^+^ cells from hFK were mixed with dissociated hFK cells and cultured for 7 days in an ex vivo dissociation‐reaggregation procedure 36. Confocal microscopy with immunostaining confirmed that SIX2^+^CITED1^+^ cells localized within recognizable developing renal structures and contributed, for example, to the hFK populations coexpressing SIX2 and CITED1 (Fig. 7A–7F). Additionally, in some instances, we found that CM‐DiI‐labeled SIX2^+^CITED1^+^ cells expressed renal lineage markers such as WT1 (uninduced and induced cells, restricted to podocytes upon maturation; supplemental online Fig. 5A), E‐cadherin (epithelial marker, acquired during mesenchymal to epithelial transition; supplemental online Fig. 5B), aquaporin‐1 (proximal tubules; supplemental online Fig. 5C), and nephrin (podocytes; supplemental online Fig. 5D). Complete differentiation of NPs into mature renal cells in this ex vivo system is limited by the short time frame of our investigation and by the lack of addition of renal‐differentiation stimulating growth factors as opposed to other experimental systems, for example the induction of organoids/iPS 9, 10. Therefore, we proved nephrogenic potential of these cells in vitro using our previously established protocols 12.

SIX2^+^CITED1^+^ cells seeded into a 3D collagen system or induced by BMPs (BMP‐2 and BMP‐7) showed the ability to spontaneously form tubular‐like structures (Fig. 7G), undergoing mesenchymal‐to‐epithelial transition (MET) (coexpression of vimentin and cytokeratin; Fig. 7H), and expressing mature tubular markers (Fig. 7I–7L; supplemental online Fig. 5E, 5F). In addition, using our established protocol 12, we showed that SIX2^+^CITED1^+^ cells can be induced toward a podocyte‐like phenotype in vitro, as shown by their change in morphology (Fig. 7M) and expression of podocyte‐specific markers including WT1, FOXC2, synaptopodin, and podocin (Fig. 7N–7Q; supplemental online Fig. 5G–5J). Production of collagen IV α3 and α5 chains was also confirmed by Western blot upon differentiation and compared with expression in undifferentiated NPs (Fig. 7R; supplemental online Fig. 5K). Even if more specific analysis is required to further demonstrate specification and differentiation of these NPs, these preliminary results indicate that these cells can be pushed to nephrogenic commitment in vitro.

## Discussion

With the need for new tools to elucidate the molecular and cellular pathways that lead to human nephrogenesis, the possibility of characterizing NPs isolated from hFK is an important step toward improving our understanding of the mechanisms responsible for such aspects as exhaustion of the pool of NPs during development.

In an attempt to understand how the human nephrogenic niche is regulated, many laboratories have explored the use of stem cells, in particular iPSs and ESCs, to develop ex vivo tools to investigate the formation of mature renal structures 6, 7, 8, 9, 10, 26, 37, 38. These technologies have confirmed that it is possible to obtain NPs in vitro that can generate renal organoids manifesting characteristics similar to human fetal kidney. Nevertheless, these studies have not investigated the obtainment of pure SIX2^+^CITED1^+^ NPs and whether these stem cell‐generated NPs are developmentally equivalent to their in vivo counterparts from normal embryos.

The most important finding of this work is the validation of the use of RNA probes as a novel method for the labeling, identification, and isolation of live SIX2^+^CITED1^+^ NPs without the use of genetic manipulation or laborious stepwise protocols, which are needed to induce iPSs or ESCs toward the NP phenotype. We have confirmed the versatility of this system that allows the isolation of SIX2^+^CITED1^+^ cells from both hFK and hAF. Importantly, this technology can be easily extended to other tissues/organs where isolation based on intracellular markers or transcription factors is required.Taking advantage of the isolation of live cells, we were able to perform a genome‐wide profiling of human NPs. Multiple growth factors, including glial cell‐derived neurotrophic factor (GDNF) secreted by the MM, have been shown to have an essential role in controlling and stimulating UB branching 39, initiating the first stages of nephron development. In addition to the presence of *GDNF*, we found that one of the highest differentially expressed genes was Neuropeptide Y (*NPY*, 7.7 logFC; supplemental online Table 1). Interestingly, *NPY* has been previously identified as a key molecule that modulates Wolffian duct and UB budding by supporting GDNF signaling 40. This suggests that isolated NPs are characterized by the expression of genes that have an important role in stimulating UB branching in endogenous NPs in vivo. Further highlighting the important role of regulatory mechanisms of UB branching, we found in NPs the upregulation of *GREM1* (Gremlin 1), a known modulator of the UB tip outgrowth that acts through inhibition of BMP4 signaling 41. This implies that SIX2^+^CITED1^+^ cells regulate UB branching by both release of promoting molecules such as NPY and inhibitory signals including GREM1 (Figs. 3D and 4A–4D).

In addition, it is known that initiation of MET is marked by the expression of E‐cadherin 42. Interestingly, our analysis demonstrated that SIX2^+^CITED1^+^ cells express genes involved in the suppression of MET such as *SNAI2* (suppresses the activity of the E‐cadherin promoter) and *FOXC2* (inhibits the transcription of E‐cadherin, confirming the − phenotype of the isolated population 8, 43). Our observations also revealed that, within the selected population, genes that are essential for the induction, specification, and formation of more mature nephron structures such as *LHX1* (involved in the morphogenesis of renal vesicles and comma‐ and s‐shaped bodies), *WNK4* (tubule induction and specification), and *IRX* genes (loop of Henle specification) are downregulated (Fig. 3D), suggesting that these cells are not already induced toward specification 1. This “snapshot” of the transcriptional network confirmed that SIX2^+^CITED1^+^ cells are indeed enriched for genes involved in CM renewal as previously described in mice, but also allowed the identification of highly expressed genes such as NPY and GREM1, possibly suggesting a unique gene signature of human NPs.

Most of our knowledge on renal development comes from the study of rodents. However, it is widely accepted that although mammalian development shares several common traits, important differences can be found between mice and humans 2, 34, 35. A preliminary comparison of RNA‐seq data and histological analysis between the human NP populations and murine MM cells has confirmed both common traits and differences. One of the major differences was the diverse localization of the CITED1^+^ cells within the human CM. In mouse, the CITED1^+^ cells are clearly localized within the CM in close proximity of the tip of the UB 1, whereas in the human CM, CITED1 expression appears to extend further down along the edges of the UB (where usually the induced SIX2^+^ cells are found; Fig. 1B 35), probably suggesting the existence of different compartmentalization within the human MM. Remarkably, our RNA‐seq analysis (and immunostaining) identified the expression of FOXD1 within SIX2^+^CITED1^+^ cells. During mouse kidney development, FOXD1 is usually known to be highly expressed within the cortical interstitial cells surrounding the CM 1, 44, 45 and has an essential role in regulating progenitor cell differentiation 23, 44, 46, 47. Cells coexpressing both SIX2 and FOXD1 are generally not thought to exist during kidney development, but interestingly, in a fascinating work of single‐cell analysis of mouse MM, Brunskill et al. reported the sporadic presence of SIX2^+^FOXD1^+^ cells 31. Here we report the presence of SIX2^+^CITED1^+^FOXD1^+^ cells (Fig. 5C); even if more in‐depth analysis is required, we speculate that these data possibly provide evidence for multilineage priming (nephron and stroma) within the SIX2^+^CITED1^+^ cells.

In addition to the discussion above, we believe that this preliminary analysis is significant for three reasons. First, it might reveal specific molecular pathways that can optimize the differentiation of human iPSs and ESCs or enhance reprogramming to NPs (now limited to 0.87% 8) in a more efficient manner, because currently renal cell specification in vitro is achieved using growth factors that were identified in studies of mouse development. Second, this analysis might help identify surface markers that distinguish the self‐renewing fraction of the CM (specifically the SIX2^+^CITED1^+^ cells), thus favoring methods complementary to Smartflare for the direct isolation of these NPs from in vivo and in vitro systems with higher efficiency. Third, it might reveal specific important molecular signaling that can be useful to implement media conditions for the culture of NPs, possibly facilitating the maintenance of a pure nephrogenic state in vitro for many passages in culture, thus allowing the expansion of these cells in large quantities and facilitating studies ranging from cell specification and renewal to renal regeneration.

## Conclusion

In this study, we have validated the use of a novel tool for the isolation of live human NPs without the use of genetic manipulation or laborious inducing protocols. We also provide the first characterization of NPs isolated from human fetal kidneys while demonstrating the possibility of culturing these cells in a reproducible manner. Therefore, not only does this work provide the basis for the development of novel strategies for the isolation of NP, but this in vitro system will also facilitate studies of human renal development and provide a novel tool for renal regenerative purposes.

## Author Contributions

S.D.S.: conception and design, collection and/or assembly of data, manuscript writing; M.E.T.: collection and/or assembly of data, data analysis and interpretation; A.P. and M.L.‐P.: collection and/or assembly of data; S.S.: collection and/or assembly of data, manuscript writing; B.H.G.: provision of study material or patients, manuscript writing; R.E.D.F.: data analysis and interpretation; L.P.: conception and design, data analysis, final approval of the manuscript.

## Disclosure of Potential Conflicts of Interest

The authors indicated no potential conflicts of interest.

## Supporting information

Supporting Information

Supporting Information

Supporting Information

Supporting Information
