# Supplementary material for: Direct Isolation and Characterization of Human Nephron Progenitors
Source: Stem Cells Transl Med. 2016 Sep 9;6(2):419–33. doi: 10.5966/sctm.2015-0429 (PMC5442819; doi:10.5966/sctm.2015-0429)
Supplement: Supplementary file 1 — Supporting Information [file SCT3-6-419-s001.pdf]

Supplemental Data, Figures and Movie – Perin et al.

Supplementary Data

Supplementary Discussion

Gene profiling in SIX2$^+$CITED1$^+$ cells from hAF

Interestingly, when comparing the positive NP selections from three different samples of hAF (Fig. S4A-B), we found that around 21369 genes out of 65340 were in common between all samples while only 200-800 genes were exclusively expressed by any single population. This suggests that our method is effective in isolating similar cell lines and that while in culture, different samples of hAF lines maintain similar characteristics (Fig. S4C).

We found that 1325 genes were differentially expressed between the positively and negatively selected cells (LogFC>1.5; <-1.5; p-value<0.05) including genes involved in renal development with a total of 459 genes overexpressed and 866 genes underexpressed (Fig. S4D, Table S3). While expression of CITED1 was identified by RNA-seq in all the Smartflare selected AF samples, the detectable expression of SIX2 was evident only in sample A. However, SIX2 expression was confirmed by PCR (Fig. S4B), suggesting probably that sub-optimal RNA extraction and library generation occurred for sample B and C. Therefore, since the expression of SIX2 and CITED1 was confirmed by PCR (right after Smartflare selection on the same samples that were processed for RNA-seq), we were confident in including sample B and C in the current analysis.

In particular, of the genes specifically involved in nephron formation, we found that the major overexpressed gene in SIX2$^+$CITED1$^+$ cells was *GREM1* (Fig. S4E) while *WNT4, GPC3 and DCHS1* (a regulator of the number of nephron progenitors) were significantly downregulated. It is important to note that the lack of dramatic differences in nephrogenic–related DE genes in the

hAF-derived populations comes as no surprise, since hAKPC-P (from which the $SIX2^{+}CITED1^{+}$ cells are isolated) were previously enriched for a renal phenotype (Fig. 5) by selection for CD24, OB-cadherin and podocalyxin [2].

Supplementary Materials and Methods

Acquisition of hFK and hAF samples, single cell suspension and fibroblast obtainment

For the current study, 3 samples of hAF (17.1, 17.8, 18.0 GA) with normal male karyotypes and normal fetal ultrasounds (kindly donated by Dr R. Habibian, Labcorp, Monrovia, CA) were collected by amniocentesis. Written or verbal consent was not required since samples of AF were not identified and information obtained about the samples was limited to karyotype and fetal health status (45 CFR 46.102). Based on these facts, and after a detailed review, the requirement for an approval was waived by the CHLA IRB/CCI committee. From each sample, human kidney progenitor cells (hAKPC-P) were isolated as previously described [2]. A total of 14 hFK (around 17 GA) were obtained from CHLA Bank Tissue and used for all the experiments. Tissue collection for this study was approved by the Institutional Review Boards of both Children's Hospital Los Angeles and the University of Southern California. Tissue donors provided informed consent, and no Identifying Health information was collected. After digestion with 0.05% collagenase I (BD Biosciences) at 37°C for 90 minutes and elimination of erythrocytes by Blood Lysis kit (Miltenyi Biotech), single cell suspension from hFK were obtained. Mouse amniotic fluid was collected as reported by our laboratory [2-4] following standard procedures approved by CHLA IACUC. Human Lung fibroblasts were purchased from LifeLine Cell Technology and expanded with Fibrolife Media (LifeLine Cell Technology) in tissue culture dishes for up to 5 passages.

RNAseq detailed description

RNA extraction was performed immediately after FACS (passage 0) by using the Qiagen RNeasy Micro Kit following manufacturer recommendations. Low input amount of RNA was converted to cDNA using the Clontech SMARTer V3 kit. RNA amounts were estimated following analysis on an Agilent Bioanalyzer Pico RNA chip. The ERCC standards were added, with appropriate dilutions being calculated based on the estimated RNA concentrations. The Clontech protocol for cDNA production was followed according to manufacturer's instructions. Amplification cycles were estimated based on input amounts of RNA. cDNA was again visualized by Bioanalyzer to confirm size and amounts, then sonicated on a Covaris S2 according to Clontech recommended conditions. DNA libraries were constructed using the Kapa Hyper prep kit and NextFlex adapters (Bioo Scientific). Libraries were visualized by bioanalyzer analysis and quantified by qPCR (Kapa library quantification kit) prior to application on an illumina NextSeq 500. RNA sequencing short reads were analyzed with FastQC (bioinformatics.babraham.ac.uk, 2015) and were of acceptable quality. Adapter sequences and sequences with low Phred quality scores were removed with Trimmomatic [5]. Reads were aligned to the Gencode version 22 human genome [6]corresponding to the GRCh38.p2 human genome (Genome Reference Consortium) and supplemented with sequences for the External RNA Controls Consortium synthetic RNA controls (ERCC ExFold, Ambion) [7]. Data from the mouse single cell analysis [8], were downloaded in the SRA format of the NCBI GEO database, accession GSE59127, processed similarly to the human samples and aligned to the Gencode version M6 mouse genome corresponding to the GRm38.p3 mouse genome (Genome Reference Consortium). All reads were aligned using the RNA-star short read aligner with the ENCODE recommended parameters [9]. Read counts per transcript were obtained using the HTSeq-count python script [10]. Reads per kilobase per million mapped reads (RPKM) were generated using the edgeR [11] R/Bioconductor software package [12]. Relative log expression graphs and principle component

graphs were generated using the plotting functions of the EDASeq R/Bioconductor software package [13]. Differential gene expression was analyzed using the ERCC ExFold probes with the Remove Unwanted Variation R/Bioconductor software package [14] combined with edgeR [15]. GO enrichment analysis was performed using the GOstats R/Bioconductor software [16]. Comparisons between human and mouse were facilitated by the use of the EnsemblCompara web service of Ensembl biomart which allowed correspondence between mouse and human genes and a metric for homology [17]. Clustering and plotting of heatmaps was performed with the R software package 'gplots'. Directed acyclic graphs with data generated from the package GOstats were plotted using the 'Rgraphviz' software package. Smear plots of RNA seq data were plotted with the ggplot2 software package [18] enhanced with the RColorBrewer package.

For the purpose of comparing RNA-seq data between human and mice, single cell analysis were stratified by their expression for *SIX2, CITED1* and *FOXD1.* Specifically, cells were grouped as follows: 1) $SIX2^{+}CITED1^{+}FOXD1^{+}$ 2) $SIX2^{+}CITED1^{+}FOXD1^{-}$ 3) $SIX2^{+}CITED1^{-}FOXD1^{-}$ 4) $SIX2^{-}CITED1^{-}FOXD1^{-}$ 5) $SIX2^{+}CITED1^{+}FOXD1^{-}$ 6) $SIX2^{-}CITED1^{+}FOXD1^{-}$ 7) $SIX2^{-}CITED1^{+}FOXD1^{+}$ 8) $SIX2^{-}CITED1^{-}FOXD1^{+}$.

Enrichment for NP genes from hAKPC-P *using negative selection for induced/differentiative markers*

As suggested by Brown et al. [1] we used a modified (no use of anti-erythroid marker, since cells in culture were already depleted for this cell type) negative selection for enrichment of NP markers. Following trypsinization of hAKPC-P, cells were blocked with 1X human IgG for 15 minutes and stained with CD140a, CD326, CD105 antibodies for 1 hour on ice. After washing steps, cells were sorted using a FacsAria flow cytometer (BD Biosciences) and expanded for

further analysis. RNA extraction and PCR analysis were performed as reported in the Experimental Procedure section in the main Manuscript.

Clones generation

Clones from both populations were obtained by limiting dilution immediately after sorting (passage 0): briefly, a total of 300-400 cells were singularly plated in each well of 96-multiwell plates. 4 plates were prepared for each hAF or hFK derived samples. Cultures were examined daily for the appearance of colonies. Wells containing more than one colony were not considered. All the clones that reached confluence were detached with 0.05% trypsin-0.01% EDTA (Sigma, St Louis, MO, USA) and each of them plated in four wells (replicas) of a 24-multiwell plate.

Dissociation/reaggregation Assays

hFK cells were mixed in a 10:1 ratio with either hAF- or hFK-derived $SIX2^{+}/CITED1^{+}$ cells, at passage 5 after selection, previously labeled with CM-DiI (Invitrogen) following standard protocols [19]. Cells were transferred onto polycarbonate membrane (3 µm pore size) at the air-liquid surface in DMEM growth medium in a 24 well plate for 7 days. After 7 days of culture, the kidney explants were fixed with 4% PFA. CM-Dil-labeled cells were visualized by immunofluorescence microscopy after immunostaining.

Wnt9B induction, 3D collagen experiments and podocyte induction

Induction of hFK cells toward differentiation was performed by adding Wnt9b (0.4 µg/ml) and BMP7 (0.05 µg/ml) to the culture media for 7 days. Cells were then harvested, fixed and flow cytometry analysis to evaluate expression of SIX2 and CITED1 was performed as previously described.

Induction into tubular-like cells was performed by seeding the cells at passage 5 after selection into a three dimensional collagen layer using the EMD-Millipore 3D collagen assay kit, following the manufacturer instructions. Cells were placed into 24 well plates and cultured for up to 21 days with RPMI 1640, 10% ES-FBS and 1% penicillin-streptomycin. Podocyte induction was performed as previously published [2]. Briefly, differentiation was performed by culturing the cells on collagen I coated plates in VRADD media [RPMI-1640 supplemented with 10% FBS, 1% antibiotic, 1,25(OH)2D3 (100 nM), ATRA (1 µM), dexamethasone (100 nM), 1× insulin-transferrin-selenite (ITS)] for up to 30 days.

List of Antibodies

**Antibody**	**Company**	**Catalogue Number**	**Dilution**	**Antigen Retrieval (whenever applicable)**
SIX2	Proteintech	11562-1-AP	IF: 1:100<br>FC:0.78 ug/ml	Low pH
CITED1	Novus Bio	H0004435-MO3	IF: 1:200<br>FC: 1 ug/ml	Low pH
GREM1	Santa Cruz	SC-28873	IF: 1:200	Low pH
VCAM1	Santa Cruz	SC-8304	1:50	Low pH
FOXD1	Abcam	ab129324	1:300	Low pH
NPY	Novus Bio	NBP1-46535	1:200	Low pH

LHX1	Fitzgerald	10R-7019	FC: 1:100	NA
OSR1	Thermo Scientific	PA5-17297	FC: 1:100	NA
CALBINDIN	Abcam	ab11426	1:100	Low pH
PAN CYTOKERATIN	Abcam	ab6401	1:100	Low pH
VIMENTIN	Abcam	ab92547	1:100	Low pH
PODOCIN	Abcam	ab50339	1:100	Low pH
CD105	Invitrogen	MHCD10520	FC: 1:200	NA
CD140a	Invitrogen	A15718	FC: 1:200	NA
CD326	Invitrogen	A15755	FC: 1:200	NA

List of Primers

**Primer**	**Sequence**	**Annealing Temperature**	**bp**
SIX2	GCCGAGGCCAAGGAAAGGGAG<br>GAGTGGTCTGGCGTCCCCGA	63.5	131
CITED1	AGGATGCCAACCAAGAGATG<br>TGGTTCCATTTGAGGCTACC	55.4	108
HOXA11	CTCCTACTCCTCCAACCTGC<br>AACTGGTCGAAAGCCTGTGG	56.5	294
SALL1	AGTTCTGGCAACACCATCAT<br>GGTGAGGACGATGATGAGAC	63.5	136
EYA1	GCTTAGGTCCTGTCCGTT<br>GTTCATCTGGGACTTGGA	53.8	211
ACTB	AGAAAATCTGGCACCACACC<br>CTCCTTAATGTCACGCACGA	60	410

Supplementary References

1. Brown AC, Muthukrishnan SD, Guay JA et al. Role for compartmentalization in nephron progenitor differentiation. **Proceedings of the National Academy of Sciences of the United States of America**. 2013;110:4640-4645.
2. Da Sacco S, Lemley KV, Sedrakyan S et al. A novel source of cultured podocytes. **PloS one**. 2013;8:e81812.
3. Da Sacco S, De Filippo RE, Perin L. Amniotic fluid as a source of pluripotent and multipotent stem cells for organ regeneration. **Current opinion in organ transplantation**. 2011;16:101-105.
4. Sedrakyan S, Da Sacco S, Milanesi A et al. Injection of amniotic fluid stem cells delays progression of renal fibrosis. **Journal of the American Society of Nephrology : JASN**. 2012;23:661-673.
5. Bolger AM, Lohse M, Usadel B. Trimmomatic: a flexible trimmer for Illumina sequence data. **Bioinformatics**. 2014;30:2114-2120.
6. Harrow J, Frankish A, Gonzalez JM et al. GENCODE: the reference human genome annotation for The ENCODE Project. **Genome research**. 2012;22:1760-1774.
7. Kralj JG, Salit ML. Characterization of in vitro transcription amplification linearity and variability in the low copy number regime using External RNA Control Consortium (ERCC) spike-ins. **Analytical and bioanalytical chemistry**. 2013;405:315-320.
8. Brunskill EW, Park JS, Chung E et al. Single cell dissection of early kidney development: multilineage priming. **Development**. 2014;141:3093-3101.
9. Dobin A, Davis CA, Schlesinger F et al. STAR: ultrafast universal RNA-seq aligner. **Bioinformatics**. 2013;29:15-21.

10. Anders S, Pyl PT, Huber W. HTSeq--a Python framework to work with high-throughput sequencing data. **Bioinformatics**. 2015;31:166-169.
11. Robinson MD, McCarthy DJ, Smyth GK. edgeR: a Bioconductor package for differential expression analysis of digital gene expression data. **Bioinformatics**. 2010;26:139-140.
12. Dudoit S, Gentleman RC, Quackenbush J. Open source software for the analysis of microarray data. **BioTechniques**. 2003;Suppl:45-51.
13. Risso D, Schwartz K, Sherlock G et al. GC-content normalization for RNA-Seq data. **BMC bioinformatics**. 2011;12:480.
14. Risso D, Ngai J, Speed TP et al. Normalization of RNA-seq data using factor analysis of control genes or samples. **Nature biotechnology**. 2014;32:896-902.
15. Robinson MD, Smyth GK. Moderated statistical tests for assessing differences in tag abundance. **Bioinformatics**. 2007;23:2881-2887.
16. Falcon S, Gentleman R. Using GOstats to test gene lists for GO term association. **Bioinformatics**. 2007;23:257-258.
17. Vilella AJ, Severin J, Ureta-Vidal A et al. EnsemblCompara GeneTrees: Complete, duplication-aware phylogenetic trees in vertebrates. **Genome research**. 2009;19:327-335.
18. Wickham H. Ggplot2 : elegant graphics for data analysis. New York: Springer; 2009:viii, 212 p.
19. Hendry CE, Vanslambrouck JM, Ineson J et al. Direct transcriptional reprogramming of adult cells to embryonic nephron progenitors. **Journal of the American Society of Nephrology : JASN**. 2013;24:1424-1434.

Supplemental Figures

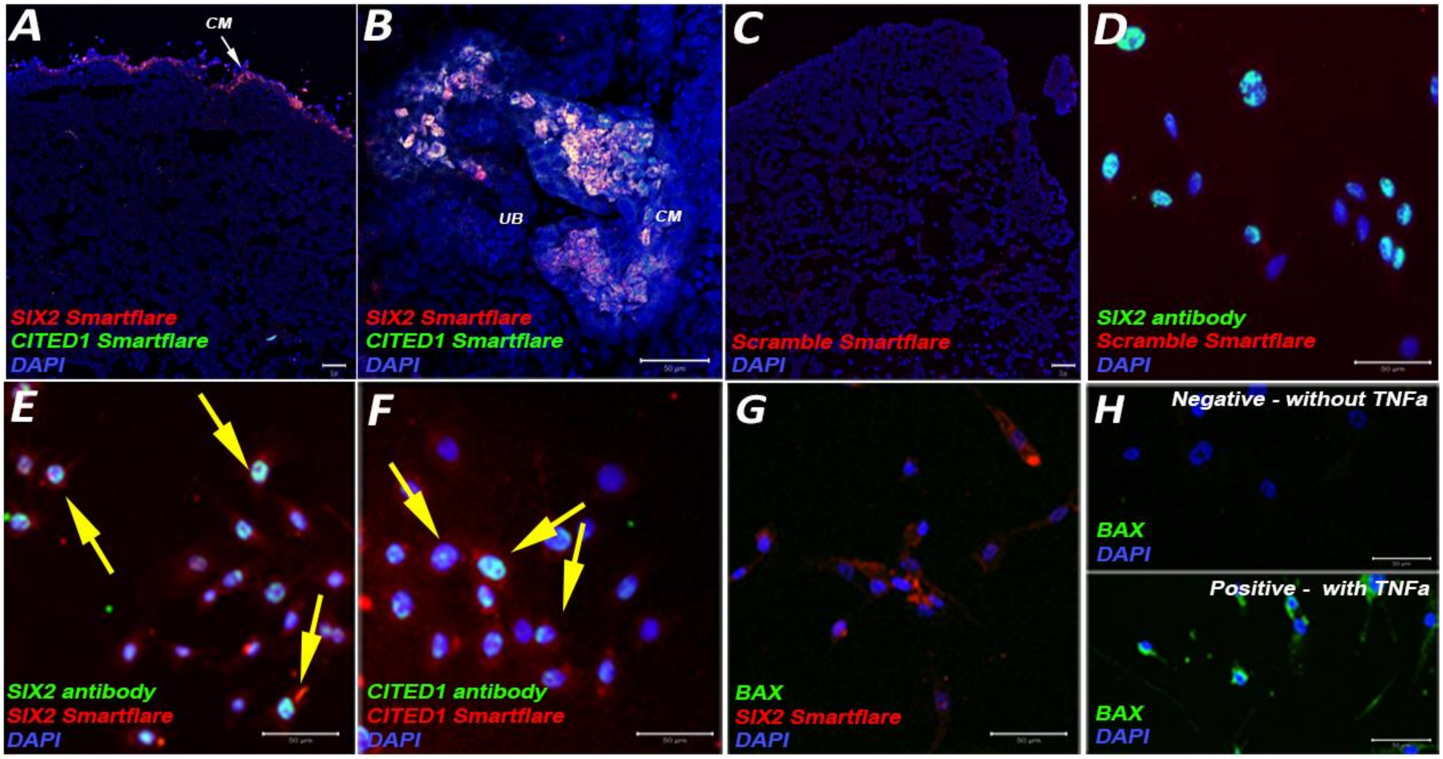

FigureS 1: In vitro Smarflare validation

A-B. Confocal images showing localization, within the CM and in proximity of the UB branching (arrow) of SIX2 (red) and CITED1 (green) Smartflare probes in renal slices (**A**, 10x, **B**, 20x; Nuclei stained blue, DAPI, scale bar: 50μm). **C-D.** Confocal images of scramble Cy5 Smartflare in renal slices (**C,** 10x) and in vitro cultured $SIX2^{+}CITED1^{+}$ cells (**D,** 40x, SIX2 antibody: green, scale bar: 50μm) confirming specificity of the signal and absence of background signal. Nuclei stained blue, DAPI. **E-F** Overlapped expression of the SIX2-Cy5 probe (**E**, red; arrow: RNA in perinuclear zone, 40x, scale bar: 50μm) and CITED1-Cy3 probe (**F**, red; arrow: RNA in perinuclear zone, 40x, scale bar: 50μm) with the SIX2 (**E**, green; arrow) and CITED1 (**F**, green; arrow) proteins in isolated $SIX2^{+}CITED1^{+}$ cells after 24h of culture. Nuclei stained blue, DAPI. **G-H** Negative Bax staining (green) of $SIX2^{+}CITED1^{+}$ cells incubated with SIX2 RNA probe (**G,** 20x, scale bar: 50μm). **H.** Immunostaining for BAX (green) in $SIX2^{+}CITED1^{+}$ cells (negative control, top panel) or stimulated with TNF-α (positive control, lower panel), 20x; Nuclei stained grey, DAPI, scale bar: 50μm.

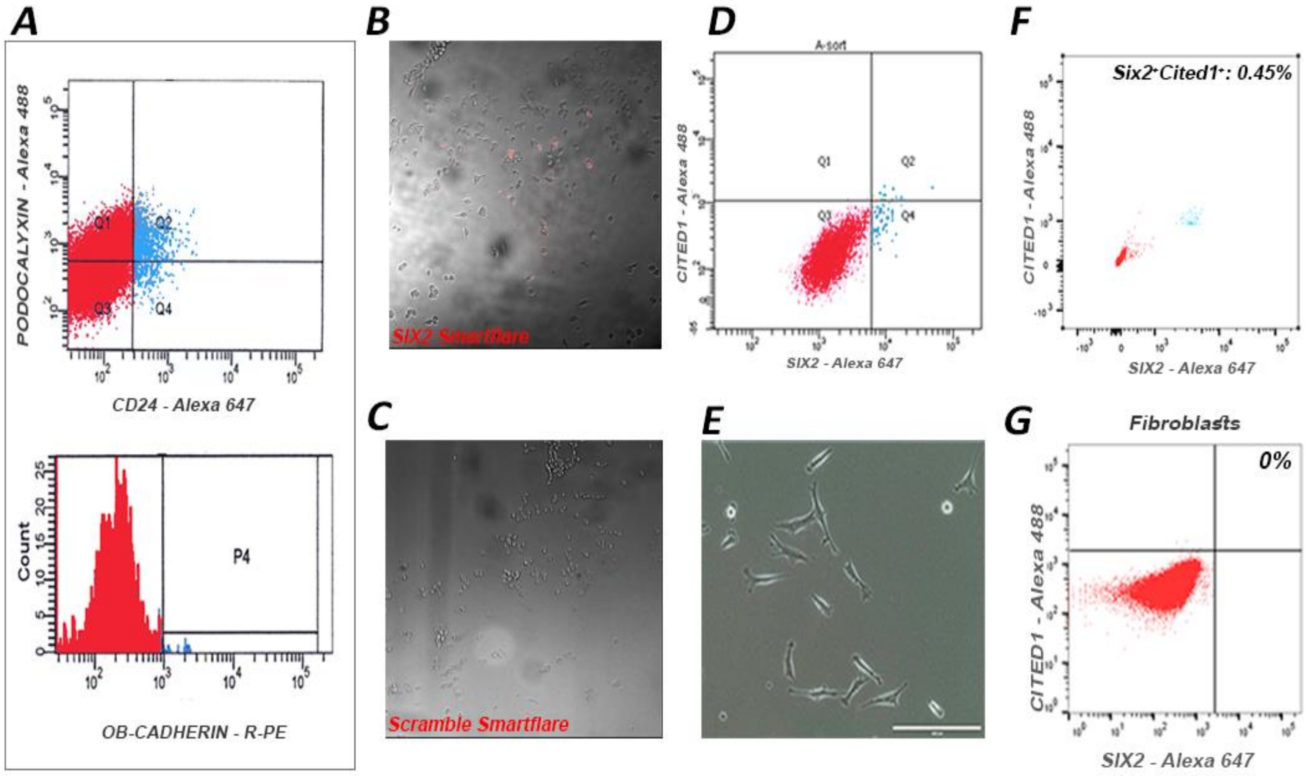

Figure S2: Isolation of SIX2^{+}CITED1^{+} cells from hAF

A. FACS sorting of hAKPC-P cells (0.4-0.5% of total population) from AF samples between 17 and 18 GA. **B-C.** Over-imposed immunofluorescence and bright field images confirming expression of SIX2 detected by Cy5-Smartflares in hAKPC-P **(B,** 10x, scale bar: 50μm**)** while no signal is detected on the same population when using scramble Cy5- Smartflares (**C,** 10x, scale bar: 50μm). **D-E** SIX2+CITED1+ cells isolated by FACS sorting with the use of RNA probes represent 0.2-0.3% of hAKPC-P population at passage 20 (**D**) and present a fibroblastoid shape (**E**, 20x). **F.** FACS analysis of hAKPC-P (passage 20) showing cells positive for SIX2 and CITED1 (0.45%). **G.** FACS sorting showing that fibroblasts resulted negative for RNA probes (SIX2-Cy5 and CITED1-Cy3) sorting.

A

Sample (hFK)	Total Reads	Unique Paired	Unique Single	Total unique	%	LW Depth
Sample 17.0 Six2+Cited1+	135827426	16837359	59933116	93607834	68.9%	22.05
Sample 17.0 negative	131965998	14129944	45696290	73956178	56.0%	17.42
Sample 17.2 Six2+Cited1+	114367594		78644414	78644414	68.8%	18.52
Sample 17.2 negative	105423864		77723508	77723508	73.7%	18.30
Sample 17.5 Six2+Cited1+	107895755		75095125	75095125	69.6%	17.69
Sample 17.2 negative	104225096		76547820	76547820	73.4%	18.03

B

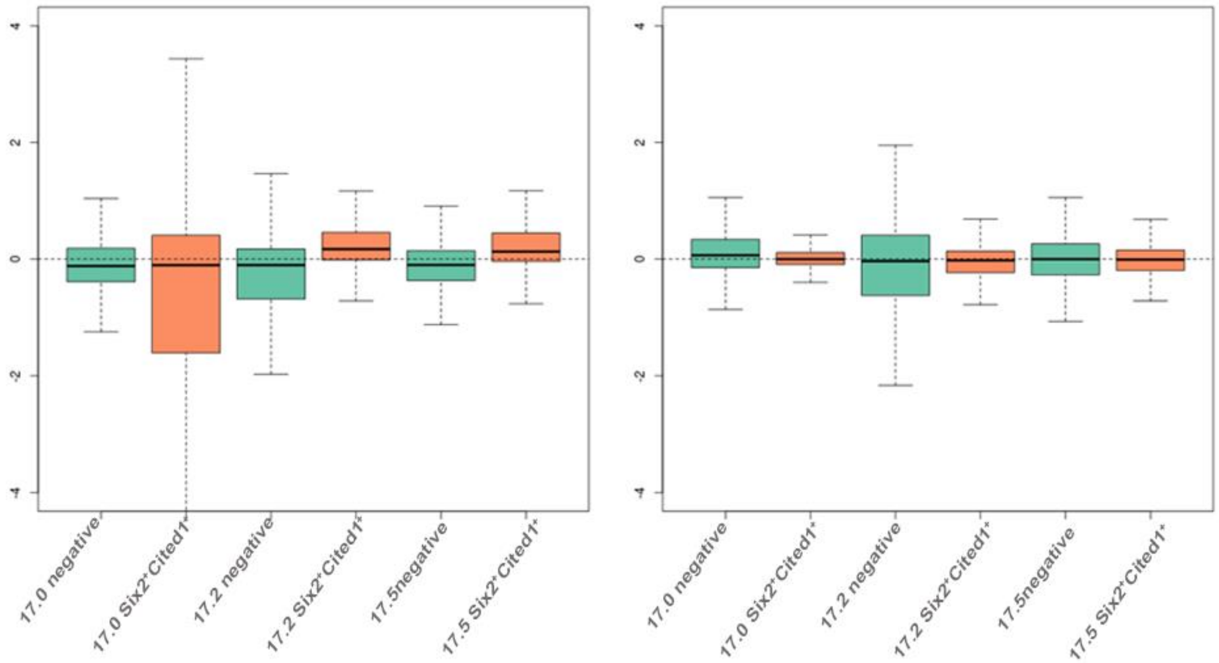

Figure S3: Gene profiling of SIX2$^+$CITED1$^+$ cells from hFK

A. Statistics for RNA-seq analysis on hFK populations. Over 70 million unique reads on average were obtained for each sample out of more than 100 million total, corresponding to 56-73% of the total reads. Lander-Waterman calculation is most likely underestimated as the gencode v22 contained all of the alternate scaffolds and assemblies, thus inflating the transcriptome length. **B.** Boxplots of RLE for unnormalized and normalized counts for hFK derived selections. Orange: SIX2$^+$CITED1$^+$ cell fractions; green: negative fractions. After normalization RLE distributions were centered around zero and confirmed to be as similar as possible to each other after normalization.

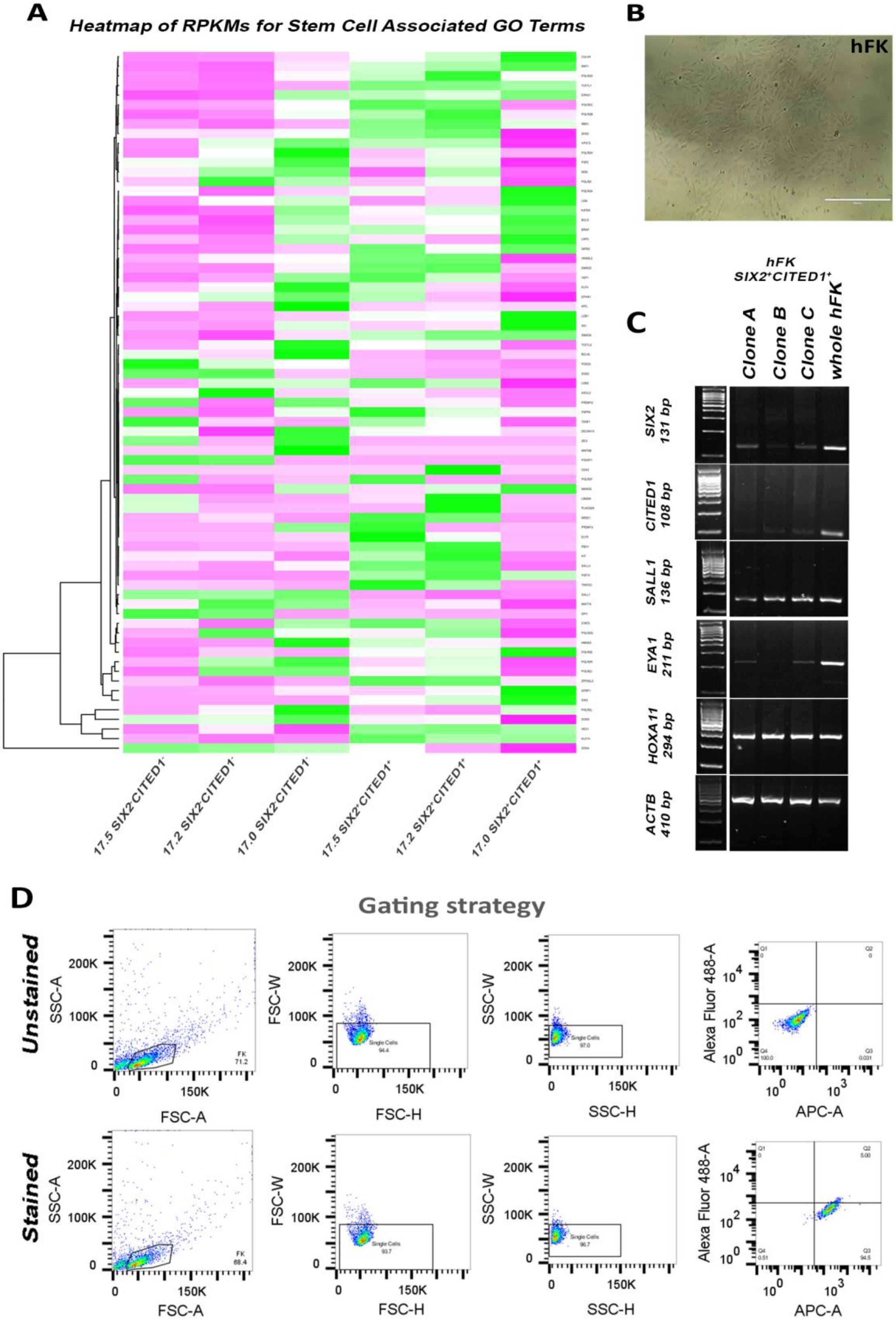
A
Heatmap of RPKMs for Stem Cell Associated GO Terms
17.5 SIX2⁻CITED1⁻
17.2 SIX2⁻CITED1⁻
17.0 SIX2⁻CITED1⁻
17.5 SIX2⁺CITED1⁺
17.2 SIX2⁺CITED1⁺
17.0 SIX2⁺CITED1⁺
B
hFK
C
hFK SIX2⁺CITED1⁺
Clone A
Clone B
Clone C
whole hFK
SIX2 131 bp
CITED1 108 bp
SALL1 136 bp
EYA1 211 bp
HOXA11 294 bp
ACTB 410 bp
D
Gating strategy
Unstained
Stained
SSC-A
FSC-A
FSC-W
FSC-H
SSC-W
SSC-H
Alexa Fluor 488-A
APC-A
FK
71.2
68.4
Single Cells
94.4
93.7
97.0
96.7
Q1
Q2
Q3
Q4
100.0
0.031
5.00
0.51
94.5
200K
100K
150K
0

Figure S4: Clonality, pluripotency markers and maintenance of competency

A. Heatmap showing relative expression (measured in RPKM) of stem cell related genes in $SIX2^{+}CITED1^{+}$ cells from hFK. **B.** Representative picture showing the morphology of clonal cells derived by serial dilution from both $SIX2^{+}CITED1^{+}$ cells from hFK in culture at passage 0 (colony formation), 20x. **C.** Gene expression analysis of clonal population derived from hFK revealed that all clones were expressing EYA1 (211 bp), SIX2 (131 bp) , CITED1 (108 bp) , HOXA11 (294 bp) and SALL1 (136 bp), confirming their traits as NP. Housekeeping gene: ACTB (410 bp). **D.** Live cells were first gated based on forward and side scatter and dead cells were excluded from the analysis. Further gating was performed to remove duplets based on FSC-W/FSC-H and SSC-W/SSC-H. Gating for positive cells was performed to exclude all events occurring in unstained cells for each channel (Alexa-488, APC). Gating was performed following the same criteria but independently for each sample to reflect differences between the analyzed populations.

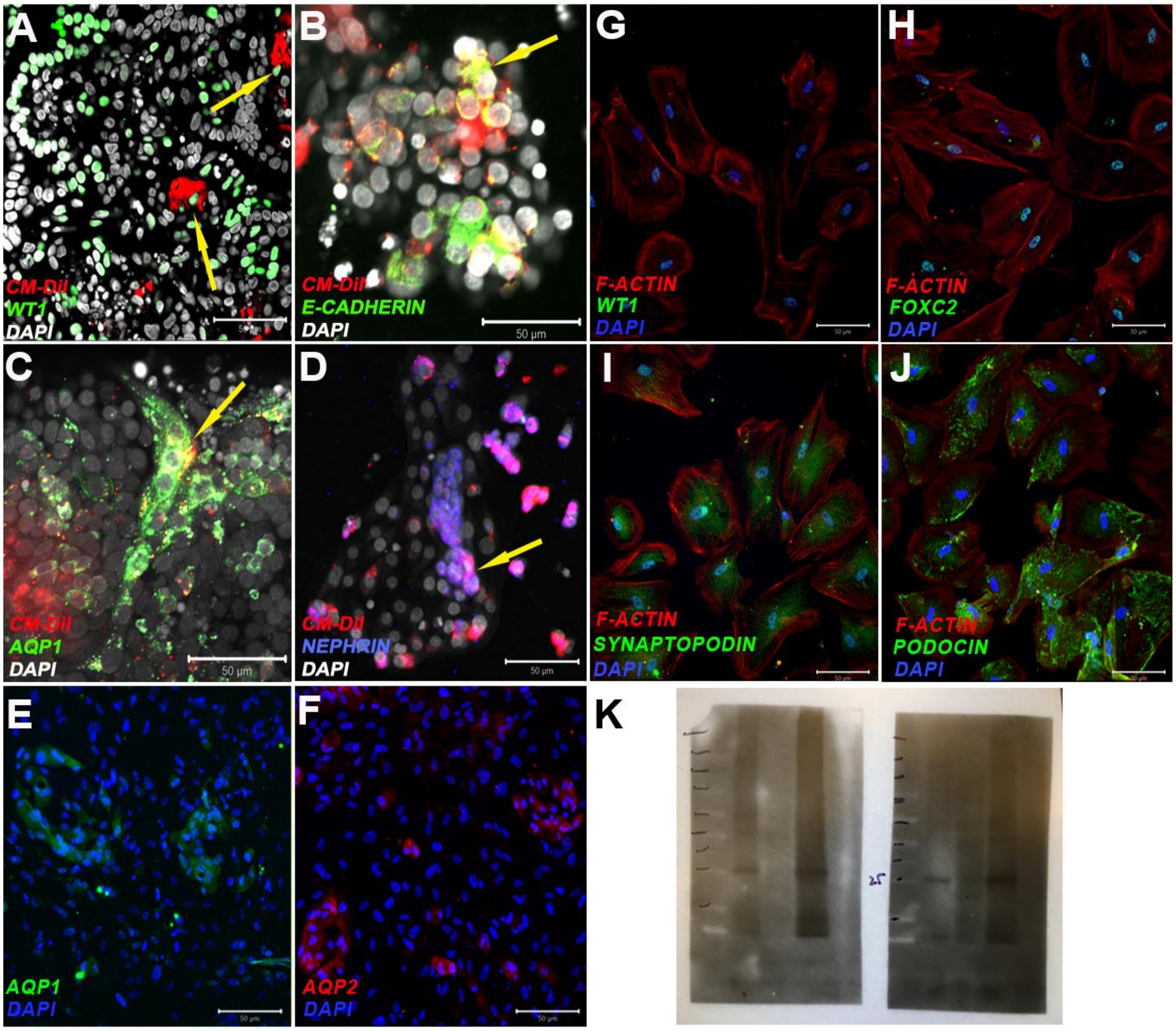
A
CM-DiI
WT1
DAPI
B
CM-DiI
E-CADHERIN
DAPI
50 µm
G
F-ACTIN
WT1
DAPI
H
F-ACTIN
FOXC2
DAPI
C
CM-DiI
AQP1
DAPI
50 µm
D
CM-DiI
NEPHRIN
DAPI
I
F-ACTIN
SYNAPTOPODIN
DAPI
J
F-ACTIN
PODOCIN
DAPI
E
AQP1
DAPI
F
AQP2
DAPI
K
25

Figure S5: SIX2^{+}CITED1^{+} cells from hFK: in vitro induction

A. Confocal images of CM-Dil labeled SIX2^{+}CITED1^{+} cells (surface marker, red) from hFK showing co-localization (arrows) staining with WT1 (nuclei, green, **A**), co-localization (yellow, arrows) with E-cadherin (surface marker, green, **B**), co-localization (yellow, arrows) with aquaporin-1 (surface marker, green, **C**) and co-localization (magenta, arrows) staining with nephrin (surface marker, blue, **D**). Nuclei stained grey, DAPI, 20x, scale bar: 50μm. **E-F.** Immunostaining of SIX2+CITED1+ cells from hFK upon induction toward tubular differentiation by addition of BMP-2 and BMP-7 to the culture media. Partially organized tubular-like structures expressing either AQP-1 (**E**) and AQP-2 (**F**) were found. Nuclei stained blue, DAPI, 10X, scale bar: 50μm. **G-J.** Immunostaining of SIX2+CITED1+ cells from hFK after podocyte differentiation confirming expression of WT1 (**G**, green), FOXC2 (**H**, green), SYNAPTOPODIN (**I**, green), PODOCIN (**J**, green). Phalloidin staining (red) identifies the actin cytoskeleton; nuclei stained blue, DAPI, 10x, scale bar: 50μm. **K.** Full membrane for the Western Blot analysis for collagen IV alpha 3 and alpha 5 chains in differentiated cells compared to undifferentiated cells and fibroblasts (negative control). Positive control: human kidney (25kDA, monomeric form; 50kDA, dimeric form).

Supplemental Movie 1: Representative animation of confocal microscopy Z-stack showing expression of SIX2 (red) and CITED1 (green) labeled with SIX2-Cy5 and CITED1-Cy3 RNA probes respectively. Co-localization (yellow) of SIX2

and CITED1 is visible in some cluster of cells surrounded by SIX2 only positive cells.
